# Causes of delayed angiosperm diversification: The photosynthetic revolution, increased opportunity costs of anti‐herbivore defenses, selection for qualitative toxins, and acceleration of plant–herbivore coevolution

**DOI:** 10.1002/ajb2.70115

**Published:** 2025-10-18

**Authors:** Thomas J. Givnish

**Affiliations:** ^1^ Department of Botany University of Wisconsin‐Madison Madison WI USA

**Keywords:** anti‐herbivore defenses, coevolution, different defenses in herbs vs. woody plants, opportunity costs, photosynthetic revolution, species diversification

## Abstract

Why did it take so long for angiosperms to diversify after they arose? Here I consider the indirect but potentially crucial impact of the “photosynthetic revolution” on plant–herbivore coevolution. Increased vein density in fossil leaves implies a doubling in photosynthesis 125–100 million years ago. Higher photosynthetic rates increase the opportunity cost of anti‐herbivore defenses, favoring shifts to chemically diverse, low‐cost, low‐molecular‐weight qualitative toxins (e.g., alkaloids) from chemically stereotyped, high‐cost, high‐molecular‐weight quantitative toxins (e.g., tannins). Given the greater functional significance of incremental changes in defensive compounds of lower molecular weight, shifts to qualitative toxins should accelerate plant‐herbivore coevolution and species diversification. The large genome and cell sizes of ferns and gymnosperms should drive lower rates of coevolutionary diversification by decreasing vein density and photosynthetic rates; high vein density found in many euasterids, eurosids, and monocots should drive higher diversification rates. This theory might also explain the general restriction of qualitative toxins to herbaceous plants, given the higher photosynthetic rates of herbs vs. woody plants. Lower hydraulic limitation and selection for small genomes in short, fast‐growing, short‐lived plants should foster evolution of small cells, fine vein networks, high leaf N levels and photosynthetic rates, reliance on qualitative toxins, and high speciation rates.

Angiosperms are by far the most diverse and ecologically dominant clade of land plants, with 295,000–400,000 species (Govaerts, 2001; Christenhusz and Byng, 2016). Their diversification is also considered a key driver of overall terrestrial species diversity, with positive effects on fungi, bacteria, pollinators, seed dispersers, and especially herbivorous insects and the predatory insects, other invertebrates, and vertebrates that feed on them (Farrell, 1998; Moreau et al., 2006; McKenna et al., 2009; Benton et al., 2022; Peris and Condamine, 2024; Wu et al., 2024). Angiosperm diversification also appears to have helped foster the rise of temperate and tropical forests (Wang et al., 2009; Boyce et al., 2010), the diversification of understory and epiphytic ferns (Schneider et al., 2004; Schuettpelz and Pryer, 2009), and the decline of conifers (Condamine et al., 2020).

The timing of the initial diversification of angiosperms—the crown age of flowering plants—is debated but appears most likely to be at least 150 million years ago. Estimates based on fossil‐calibrated DNA sequence data range from 139 to 266 Mya (Bell et al., 2010; Silvestro et al., 2015; Salomo et al., 2017; Li et al., 2019; Magallón et al., 2015, 2019; Ramírez‐Barahona et al., 2020; Yang et al., 2020; Sauquet et al., 2022). Estimates based directly on fossils are generally much younger, with the earliest unequivocal records of angiosperms being pollen from ≤140 Mya (Magallón et al., 2015; Coiro et al., 2019). However, Silvestro et al. (2021) used Bayesian approaches to show that known fossil ages alone point to an angiosperm crown age of 154–255 Mya, largely eliminating the conflict between fossil‐ and DNA‐based estimates while leaving substantial uncertainty about each.

One of the great mysteries of angiosperm evolution is why they did not rapidly diversify until long after the rise of their defining characteristics—such as flowers, seeds enclosed in ovules, closed carpels, rapid fertilization, and abundant xylem vessels (also in Gnetales)—which must have occurred no later than their crown age. Large numbers of flowering plant lineages only appeared after 120 to 80 Ma, at least 30 to 70 Ma after they acquired those traits and began to diversify (Sanderson and Donoghue, 1994; Barba‐Montoya et al., 2018; Zuntini et al., 2024). Based on fossil‐calibrated molecular phylogenies, the highest rates of net species diversification occurred only in the last 85 Ma, concentrated sporadically across orders, families, and genera of flowering plants while the average net diversification rate remained roughly constant (Magallón et al., 2015, 2019). High levels of species richness are also scattered among more recently derived orders (Soltis et al., 2019). Dimitrov et al. (2023) found a sharp rise in net species diversification rate in angiosperms ca. 10 Ma after their crown age, corresponding to low species numbers in the ANA grade (Amborellales, Nymphaeales, Austrobaileyales), Chloranthales, and (to a lesser extent) the magnoliids. Based on fossils alone, angiosperms show a 20‐ to 75‐Ma lag between their initial appearance to their climb to ecological dominance between 120 and 65 Ma (Lidgard and Crane, 1988, 1990; Benton et al., 2022), starting in the tropics and then spreading to higher latitudes (Crane and Lidgard, 1989). As with almost all major lineages, angiosperm diversification was punctuated by mass extinctions 66 Mya, caused by the Chicxulub asteroid impact (Nichols and Johnson, 2008; Wilf et al., 2023). Many angiosperm families and orders, however, survived this event and diversified subsequently. Despite fossil evidence of widespread angiosperm extinction, phylogenetic analyses by Thompson and Ramírez‐Barahona (2023) found no signature of mass extinction. Givnish et al. (2018), however, documented a secondary burst of monocot family diversification after the Cretaceous, which they attributed to competitive release following mass extinction of several ecologically dominant and speciose lineages.

What might account for the lags both in angiosperm diversification and in their rise to ecological dominance, with the occurrence of the highest net rates of species diversification only in the last 85 Ma? Clearly the characters defining the angiosperm clade were not involved, at least not by themselves. One possibility is the delayed evolution of other key traits that—once they appeared—drove higher net rates of species diversification. Phylogenetic analyses over the past 30 years have supported several traits as likely drivers of diversification within the angiosperms, including variation in life history, growth form, reproductive ecology, photosynthetic pathway, hydraulics, latitude, adaptive radiation in habitat, pollinators, and chemical defenses, whole‐genome duplications, lineage age, species and lineage geographic range sizes, and combinations thereof, as well as statistical artifacts that bias diversification rates upward in more recently derived clades (Dodd et al., 1999; Heilbuth, 2000; Ricklefs and Renner 1994, 2000; Givnish, 1999, 2010; Davies et al., 2004; Sargent, 2004; Kay et al., 2006; Silvera et al., 2009; Spriggs et al., 2014; Edger et al., 2015; Tank et al., 2015; Spalink et al., 2016; Givnish et al., 2014, 2015, 2018; Landis et al., 2018; One Thousand Plant Transcriptomes Initiative, 2019; Hernández‐Hernández and Wiens, 2020; Helmstetter et al., 2023; Yu and Wiens, 2024),. Higher diversification rates are associated with short life cycles, herbaceous habit, animal pollination, short‐distance seed dispersal, tropical distributions, bilateral flowers, epiphytism, fleshy fruits in shady understories, broad clade ranges, narrow species ranges, and young clade ages (Givnish, 2010). Recent angiosperm‐wide analyses showed that many bursts of accelerated speciation follow whole‐genome duplications after a time lag (Tank et al., 2015; Landis et al., 2018).

Clearly many different factors can affect speciation and net diversification rates in angiosperms. Variation in global conditions over the last 250 million years—including declining atmospheric CO_2_ levels (Foster et al., 2017) and a thermal peak in the Eocene (Judd et al., 2024)—may have also played a role. There is no evidence, however, that such variation or any of the aforementioned factors, acting alone, would have increased diversification or species numbers *across* several angiosperm lineages beginning 120 to 80 Ma ago.

## THE PHOTOSYNTHETIC REVOLUTION

What has not generally been appreciated, however, is the indirect but pervasive role that the “photosynthetic revolution” 125 to 90 million years ago (Brodribb et al., 2007; Boyce et al., 2009, 2010; Brodribb and Feild, 2010) may have played in accelerating plant–herbivore coevolution and resulting angiosperm speciation (Givnish, 2010). The photosynthetic revolution was recognized based on the 3‐fold increase in vein density per unit leaf area in fossil angiosperms starting around 125 million years ago. Given the relationships among photosynthesis per unit leaf area, vein density, and transpiration in present‐day vascular plants, the observed increase in vein density implies a more than doubling in photosynthesis per unit area and a 4‐fold increase in transpiration in angiosperms between 125 and 100 Ma ago—with no concurrent change in gymnosperms and ferns (Brodribb et al., 2005, 2007; Brodribb et al., 2007; Boyce et al., 2009; Brodribb and Feild, 2010).

Across representatives of all major lineages of vascular plants, leaf stomatal conductance—which helps determine the rate of water vapor loss from a leaf, as well as CO_2_ uptake—scales linearly with leaf hydraulic conductance, or *K*_leaf_ (Brodribb et al., 2005). Simply put, water loss is inevitably associated with photosynthesis, and the capacity to replace the water loss must scale with the rate of water loss. Across vascular plants, Brodribb et al. (2007) also showed that photosynthesis per unit leaf area scales nearly linearly with *K*_leaf_. These relationships among stomatal conductance, photosynthesis, and *K*_leaf_ are consistent with the models that minimize water loss for a given amount of photosynthesis per unit area (Cowan and Farquhar, 1977) or maximize whole‐plant carbon gain (Givnish, 1986). Sack and Frole (2006) and Boyce et al. (2009) showed that leaf hydraulic conductance *K*_leaf_ also increases with vein density (length per unit area) across representatives of vascular plant lineages. The connections of vein density to leaf hydraulic conductance and, thus, to photosynthesis are what makes it possible to estimate photosynthetic rates in fossil plants and thereby recognize the photosynthetic revolution.

Boyce et al. (2009) made three critical observations: (1) Non‐angiosperm vascular plants almost all have vein densities less than (often much less than) 5 mm mm^–2^, and these have remained stable over the last 380 Ma; (2) the ANA grade together with Chloranthales and magnoliids mostly also have low vein densities; and (3) later‐emerging angiosperm clades in angiosperms beyond these groups often had much higher vein densities, up to 13–24 mm mm^–2^ in monocots and eudicots. The photosynthetic revolution was thus almost entirely restricted to eudicots and monocots. Critically, greater vein density is also associated with higher photosynthetic rate per unit leaf mass (Sack et al., 2013) and, thus, energetic return on investment, and with higher whole‐plant relative growth rate (RGR, g g^–1^ day^–1^) (Kruger and Volin, 2006).

Brodribb and Feild (2010), Vermeij (2011), and Augusto et al. (2014) argued that higher photosynthetic rates may have accelerated speciation in angiosperms by giving them a competitive advantage over other lineages. There is surely some truth to this idea, but it is not clear how competitive advantage would lead to far greater diversity in angiosperms than previous radiations of vascular plants, translate into higher rates of formation of mating barriers, or generate ongoing waves of diversification once dominance by ferns and gymnosperms was broken. In addition, there is no clearly monotonic relationship between productivity and plant species richness in natural assemblages at several spatial scales—patches of Sonoran Desert are more diverse than far more productive redwood forests, infertile or heavily grazed grasslands are more diverse than more productive sites, and plant species richness often peaks at intermediate productivity (Whittaker, 1960, 1972; Grime, 1979; Tilman, 1988; Hautier et al., 2009; Fraser et al., 2015; Harpole et al., 2016; Eskelinen et al., 2022). Furthermore, the supposed edge of angiosperms in growth is not universal: gymnosperms can grow as fast or faster than woody angiosperms on less‐productive sites (Downing and Weber, 1984; Aerts, 1995; Reich et al., 1997a; Pretzsch, 2009; Augusto et al., 2014).

## PHOTOSYNTHETIC‐CHEMICAL EVOLUTION–COEVOLUTION RATES HYPOTHESIS

**Figure 1:**
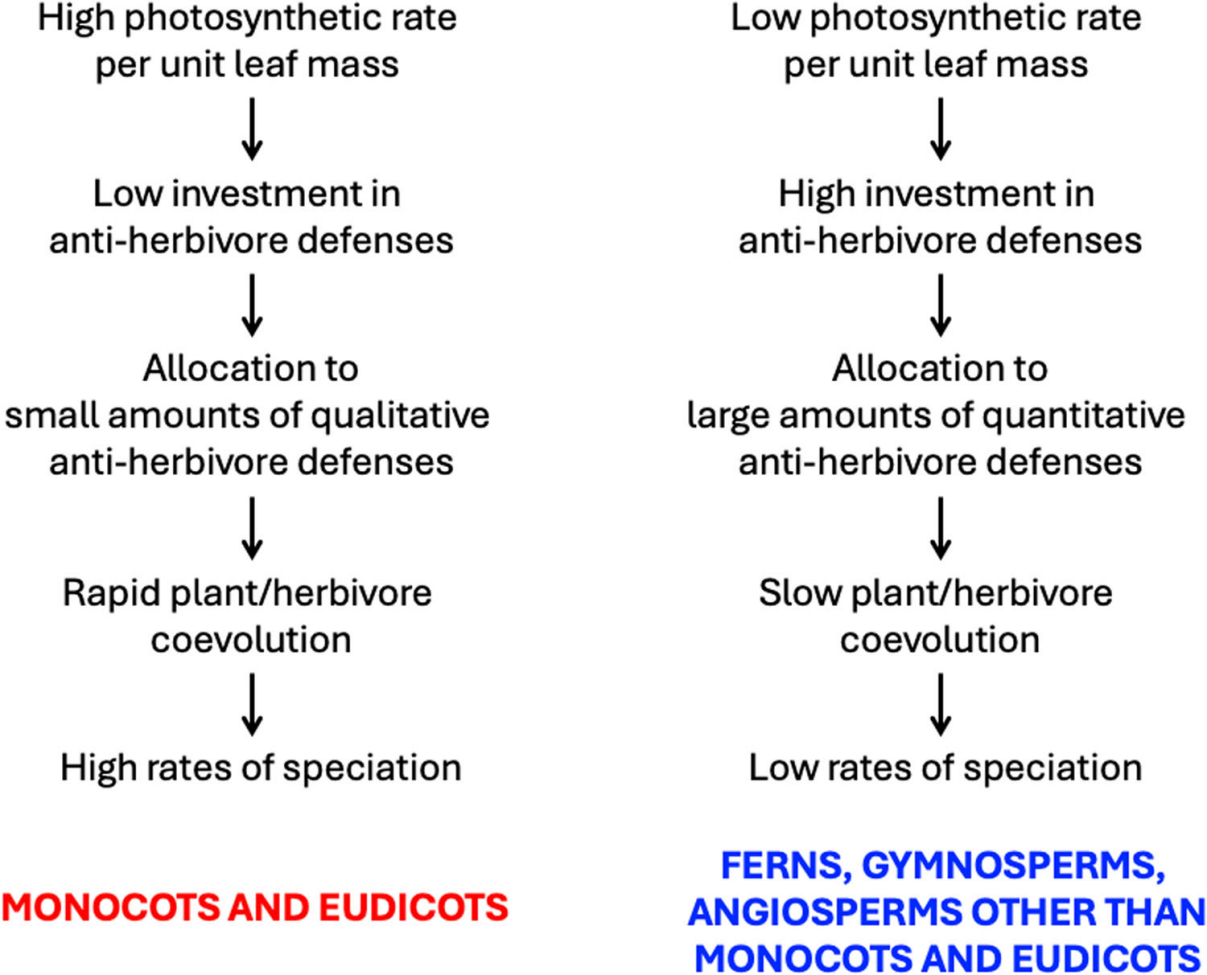
Proposed cascade of effects linking photosynthetic rates to rates of species diversification via investment in different kinds of anti‐herbivore defenses and resulting rates of chemical evolution and plant/herbivore coevolution. Low photosynthetic rates should favor low rates of species diversification in ferns, gymnosperms, and basal angiosperms; higher photosynthetic rates should favor higher diversification rates in higher angiosperms.

Here I propose that high photosynthetic rates may accelerate plant species diversification by favoring higher rates of coevolution between plants and insect herbivores, driven by selection to rely on chemical defenses of lower molecular weight that undergo faster functional evolution. This process would result in high rates of species diversification in many eurosids, euasterids, and monocots (marked by high vein densities and generally small genomes) and low rates in the ANA grade, Chloranthales, magnoliids, gymnosperms, and ferns (marked by low vein densities and often larger genomes). The logic behind this first hypothesis—substantially elaborating on an idea advanced by Givnish (2010)—involves four steps (Figure 1):

(1) Higher photosynthetic rates and whole‐plant rates of growth increase the opportunity cost of anti‐herbivore defenses, favoring a shift to lower proportional allocation to such defenses according to the resource‐availability (or growth‐rate) hypothesis (Coley et al., 1985). In this context, opportunity costs reflect the growth foregone by investing more heavily in defenses and not productive tissue; the higher the rates of photosynthesis and whole‐plant growth, the higher those costs will be. Inducible defenses can reduce opportunity costs (Heil and Baldwin, 2002; Zust and Agrawal, 2017), but bring with them the cost of herbivore damage while such defenses are being induced (Frost et al., 2008; Backmann et al., 2019). Meta‐analyses across a wide variety of plants and habitats are consistent with this hypothesis, showing that growth rates are negatively correlated with allocation to defense and positively correlated with rates of herbivore damage (Endara and Coley, 2011; Endara et al., 2023).
(2) Reduced allocation to anti‐herbivore defense should favor a shift toward *qualitative defensive compounds* (e.g., alkaloids, furanocoumarins, glucosinolates), which are effective at low concentrations (often <0.1%) and away from *quantitative defensive compounds and structures* (e.g., tannins, lignins, thick cell walls, silica inclusions), which are dosage‐dependent and often effective only at high concentrations (often >1–10% dry mass). Qualitative defenses often have low molecular weight, can generally pass through cell walls, and act as toxins against most insect herbivores, except specialists that can detoxify them or resist their effects; quantitative defenses often have higher molecular weight, cannot generally pass through cell walls, and instead act as feeding deterrents or economic defenses that complex with proteins in ruptured plant cells in the gut and make them unavailable for uptake, or generate reactive oxygen species (ROS) that cause metabolic damage (Barbehenn and Constabel, 2011).
  These distinctions between qualitative and quantitative defenses were first advanced by Feeny (1976) and Rhoades and Cates (1976) in proposing the plant‐apparency hypothesis, an alternative schema for the evolution of plant defenses. According to this hypothesis, long‐lived or abundant plants are “apparent” and will predictably be located by herbivores—including those adapted to their defenses—and so should be protected with quantitative defenses, which are effective against by most if not all herbivores. Short‐lived or rare plants are “unapparent”, so quantitative defenses can be used economically to protect against most of the few herbivores that find them. Most adherents of the plant‐apparency hypothesis have, in effect, regarded long‐lived woody plants as “apparent” and herbs as “unapparent”. Hay (2016) summarized criticisms of this hypothesis, including the difficulty of quantifying apparency, the cost of a defense based on its standing crop vs turnover rate and its composition of carbohydrates vs. mineral nutrients (e.g., N), the position of defenses along a quantitative–qualitative continuum, and the ability to make predictions for plants that possess several different types or qualities of chemical defenses. Here I am bridging the growth‐rate and plant‐apparency hypotheses to propose that selection for lower allocation to anti‐herbivore defenses should favor investment in relatively low‐cost qualitative defenses. Selection for heavier defensive allocation in slower‐growing plants should favor investment in more costly quantitative defenses or a mixture of qualitative defenses; either would protect against most/all insect herbivores.
(3) I further propose that a shift to qualitative toxins in plants with higher rates and whole‐plant growth should accelerate the rate of chemical coevolution between plants and insect herbivores because many qualitative toxins have low molecular weights (a few hundred daltons), in which changing a single atom might have a much greater functional effect than in quantitative toxins with huge molecules thousands to tens of thousands of daltons in weight. Most but not all qualitative and quantitative defensive compounds differ as expected in molecular weight (Table 1). Among quantitative defenses, condensed tannins range from 1900 to 28,000 Da; lignins and lignosulfonate polymers, from 500 to 50,000 Da; and polyphenols, 500–4000 Da. Most qualitative defenses—including alkaloids, glucosinolates, furanocoumarins, cardiac glycosides, quinic acid gallates, and non‐protein amino acids—have molecular weights of a few hundred daltons (Table 1). Polyphenolic compounds (500–4000 Da), terpenes (94–2680 Da), and flavonoids (270–750 Da) are difficult cases, often produced in high to very high concentrations typical of quantitative defenses in such tree genera as *Inga*, *Pinus*, and *Eucalyptus* (Padovan et al., 2014; Ji and Ji, 2021; Forrister et al., 2023) but having low to moderate molecular weights (500–4000 Da, 94–2680 Da, and 270–750 Da, respectively). I note that an important challenge for chemical ecology is to determine whether, in fact, the anti‐herbivore functionalities of small defensive compounds are more changed by single atomic shifts, additions, or subtractions than larger compounds, as expected on general principles.
(4) The acceleration of chemical coevolution between plants and insect herbivores should increase the rates of species diversification in both, through the “radiate and release” mechanism advanced in its modern form by Ehrlich and Raven (1964): Selection should favor shifts and embellishments of defensive compounds—and the emergence of novel defenses—to escape adapted herbivores; herbivores would, in turn, be selected to overcome or evade newly evolved, successful defenses; and so in, in a never‐ending arm's race between plants and herbivores, generating plant and animal clades marked by particular defenses and counterstrategies (see also Berenbaum and Feeny, 1981; Berenbaum, 1983; Coley et al., 2019; Forrister et al., 2023; Endara et al., 2015; Agrawal and Zhang, 2021).

**Table 1:** Approximate ranges of molecular weights (MW) of quantitative vs. qualitative defensive compounds in vascular plants.

Quantitative defenses	MW (Da)	Source
Condensed tannins	1900–28,000	Aboagye and Beauchemin (2019)
Soluble tannins	500–3000	Aboagye and Beauchemin (2019)
Lignins	1000–300,000	Tolbert et al. (2014)
Polyphenolic compounds	500–4000	Haslam and Cai (1994)
Terpenes	94–2680	Hosseini and Pereira (2023)
Flavonoids	270–2100	Barnard et al. (1993); Gates and Lopes (2012)
**Qualitative defenses**		
Alkaloids	100–811	
Cardiac glycosides	387–781	Botelho et al. (2019)
Furanocoumarins	216–492	Bartnik (2024)
Glucosinolates	343–464	
Quinic acid gallates	344	
Tyrosine and related depsides	180–372	
Non‐protein amino acids	175–200	

Given that angiosperms generally have high vein densities (>5 mm mm^–2^), while the ANA grade, Chloranthales, and magnoliids—and most gymnosperms and ferns—have substantially lower vein densities (<5 mm mm^–2^: Boyce et al., 2009; Prats et al., 2024), the preceding model predicts that the early angiosperms, which were likely similar to ANA et al. as well as ANA et al. themselves should have had lower rates of species diversification than the remaining group of the “higher” angiosperms, marked by greater vein densities, photosynthetic rates, and relative growth rates. The model thus helps account for the lower diversification rate in lycophytes, ferns, and gymnosperms than angiosperms, and for at least some of the lag in angiosperm diversification, pushing it back from 140 Ma to 130–125 Ma for the crown groups of the eudicots and monocots, respectively, in the dated phylogeny of Magallón et al. (2019). It is noteworthy, however, that higher rates of diversification generally occur only in subsets of the eudicots and monocots beginning about 85 Ma based on the same phylogeny. My model makes the testable prediction that many of these fast‐diversifying lineages should be marked by sequential shifts (perhaps with short time lags) to (1) higher vein densities, (2) reliance on qualitative toxins, (3) faster rates of defensive chemistry evolution, (4) higher incidence of specialized herbivorous insect clades, and (5) greater rate of species diversification in associated clades of herbivorous insects, all in comparison with the ANA grade, Chloranthales, and magnoliids, and in sister clades with larger genomes and lower vein densities. In addition, it should be expected that (6) any sublineages within the latter group marked by higher vein densities should exhibit shifts in many of the traits predicted for the faster‐evolving angiosperm lineages, and that (7) shifts to higher diversification rates within the monocots, eurosids, and euasterids should exhibit shifts in the same traits.

## IMPLICATIONS FOR TIES OF HERBACEOUS HABIT TO QUALITATIVE DEFENSES AND HIGH RATES OF SPECIES DIVERSIFICATION

**Figure 2:**
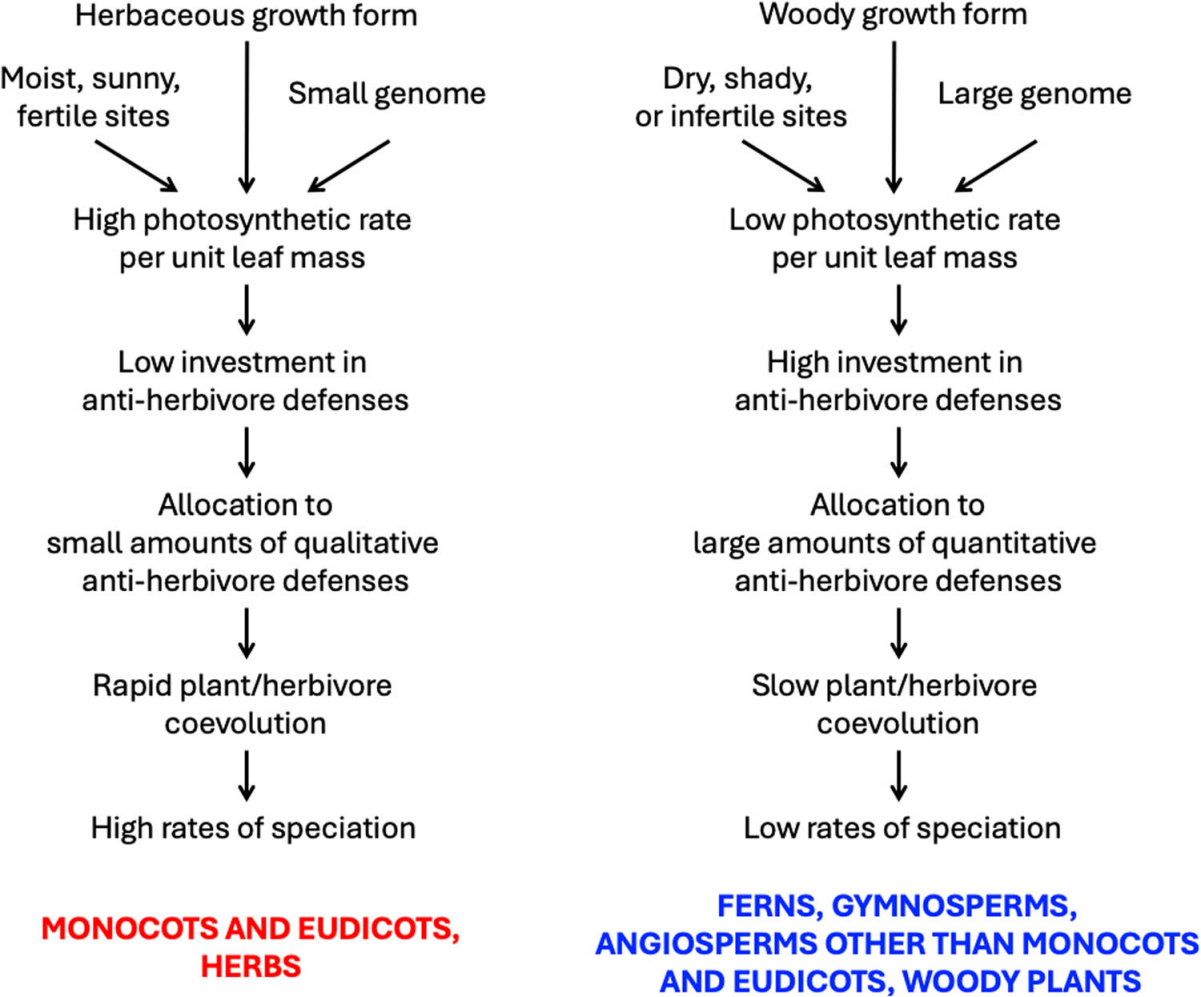
Proposed cascade of effects linking photosynthetic rates to diversification, including drivers of photosynthetic rate based on environmental conditions and organismal traits (growth form, genome size). Moist, sunny, fertile sites, herbaceous growth form, and small genomes should favor high rates of speciation; unproductive sites, woody growth form, and large genomes should favor slow rates of speciation.

If this initial model is expanded to take into account the known effects of habitat, growth form, and genome size on photosynthetic rate—and, thus, on the opportunity costs of energy allocation to anti‐herbivore defenses—it might also finally explain the strong association of qualitative defenses with the herbaceous growth form per se, highlight the driving force of historical shifts in genome size on chemical coevolution and species diversification, and make new predictions regarding higher rate of chemical coevolution and species diversification in moist, sunny, nutrient‐poor habitats (Figure 2).

Based on cost‐benefit models and empirical studies, photosynthetic rate (often on a mass basis) tends to increase toward sunnier, moister, more nutrient‐rich habitats (Givnish and Vermeij, 1976; Cowan and Farquhar, 1977; Mooney and Gulmon, 1979; Farquhar et al., 2002; Givnish, 1979, 1988, 2002; Wright et al., 2003; Givnish et al., 2014; Maire et al., 2015; Adams et al., 2019; Westerband et al., 2022; Smith et al., 2023). Herbaceous plants, on average, have higher leaf N content (N_mass_, mg g^–1^) and specific leaf area (SLA, m^2^ g^–1^) than woody plants (Ordoñez et al., 2010; Matsuo et al., 2023), implying that herbs also usually have higher photosynthetic rates per unit leaf mass (*A*_mass_, mol g^–1^ s^–1^), given that *A*_mass_ increases with both N_mass_ and SLA across a wide range of vascular plants (Reich et al., 1997b). Finally, because cell size generally increases with nuclear genome size (Price et al., 1973; Bennett, 1987; Bonner, 2006; Beaulieu et al., 2008), with this nucleotypic effect pervasive regardless of ploidy level (Francis et al., 2008), and because smaller cells allow the production of finer, denser vein networks and denser arrays of stomata, both of which enhance potential photosynthetic rates, we expect photosynthetic rates, vein density, and stomatal density to increase with decreasing genome size (Brodribb et al., 2013; Simonin and Roddy, 2018; Roddy, 2020; Théroux‐Rancourt et al., 2021).

Consequently, by including environmental and organismal drivers of photosynthetic rates, we predict increases in reliance on qualitative defensive compounds and in the rates of chemical coevolution and net species diversification in plants native to sunnier, moister, more nutrient‐rich habitats, in herbaceous vs. woody plants, and in plants with smaller genome sizes (Figure 2). A shift toward smaller genome size in angiosperms vs. other vascular plants has been argued to be a potential driver of the photosynthetic revolution, favoring increases in vein and stomatal density (Brodribb et al., 2013; Simonin and Roddy, 2018; Roddy, 2020; Théroux‐Rancourt et al., 2021). This argument is plausible, but it is important to note that the correlation between genome size and vein density is noisy. Some angiosperms outside the monocots and eudicots (e.g., several species in the ANA grade, magnoliids, and Chloranthales) have relatively small genomes *and* low vein densities, and genomes of some lycophytes—not covered in the survey by Simonin and Roddy (2018)—are very small (*Selaginella*) and or of moderate size (*Isoetes*) even though all have low vein densities (see data of Boyce et al., 2009; Pellicer et al., 2018; Wickell et al., 2021; Li et al., 2024). It is true, however, that many eurosids, euasterids, and monocots have small genomes and that species diversification rates are high in several clades scattered within these groups (Magallón et al., 2019). Gymnosperms are generally marked by lower N_mass_ and SLA, and thus presumably by lower *A*_mass_ than angiosperms (Maynard et al., 2022). Both these patterns are consistent with the theory outlined above (Figure 2). We might also expect a greater fraction of energy devoted to anti‐herbivore defense in gymnosperms vs. angiosperms, in woody plants vs. herbs, and plants with large vs. small genomes.

## CONCLUSIONS AND DIRECTIONS FOR FUTURE RESEARCH

Viewing the association of qualitative chemical defenses with herbaceous plants as being caused ultimately by their high photosynthetic rates seems far more compelling than previous explanations based on some supposed “unapparency” of herbs, regardless of their actual abundance, individual lifespan, or patch size. A critical test of this idea might be to assess the defensive chemistry, rate of chemical evolution, and rate of species diversification in herbaceous lineages in sun vs. shade, or on rich vs. poor soil, with an expected shift toward quantitative defenses and lower rates of species diversification on less productive sites. Such comparisons—extended across growth forms and conducted using phylogenetically structured comparisons—would be a means to test the third, unanticipated prediction of the model tying together photosynthetic rates, genome sizes, defensive chemistry, chemical coevolution, and species diversification rates.

In future tests of the model outlined here, five likely complications must be kept in mind. First, species diversification rates are very likely caused by multiple factors, some of which are likely to be correlated with each other across the spatially heterogenous environment and drive diversification in the same or different direction. For example, poor soils should favor low rates of photosynthesis, leading ultimately to low rates of species diversification. But poor soils should also work against fleshy fruits, fostering seed dispersal over limited distances and ultimately favoring high rates of speciation (Givnish, 2010). Similarly, shorter life cycles and more limited distances of gene flow might also lead to higher rates of speciation in herbs vs. woody plants (Givnish, 2010). Large genomes are also likely to limit plasticity and ecological niche lability and thus enhance the chance of extinction (Soto Gomez et al., 2024). As a result, tests of any proposed driver of diversification should be conducted across a wide range of lineages, ideally occupying a wide range of environmental conditions (i.e., across the entire flora of a tall island or mountain range).

Second, the model does not account for density dependence of diversification or chemical evolution. As species in a lineage accumulate in an area, especially when they make up a large share of all the species they encounter (as in an adaptive radiation on an isolated island or mountain), the rate of species diversification should slow as the range of available resources (Rabosky and Lovette, 2008; Etienne et al., 2023) or predator‐free defensive chemical space declines. Elegant studies on the tropical tree genus *Inga* by Kursar et al. (2009), Endara et al. (2022), and Forrister et al. (2023) show that coexisting species have defensive chemical profiles that differ from each other much more strongly than expected from random draws—presumably reflecting community assembly of species that avoid attack by insect herbivores that have adapted to other species’ defenses—and that species diverged in defensive chemistry at a high rate over evolutionary time. Studies of a clade like *Inga* early in its cladogenesis would presumably yield higher rates of diversification than later studies. Given the general tendency for younger clades to show higher rates of species diversification (Magallón and Castillo, 2009) and concerns that such patterns may represent statistical artifacts (Givnish, 2010), future comparisons of diversification rates should account for the estimated trajectory of diversification rate vs. time and estimated number of species present.

Third, my model does not include potential pleiotropic effects of different categories of defenses. It is possible, for example, that qualitative toxins tend to have fewer pleiotropic impacts or not affect plant metabolism as much as quantitative toxins, so that qualitative toxins might evolve faster without such constraints. Data on this issue are extremely sparse, however, and do not yet identify any general pattern. Pleiotropy and fitness constraints have been detected for quantitative defenses in *Mimulus* (Kooyers et al., 2020) and for qualitative defenses in *Boechera* (Keith and Mitchell‐Olds, 2019).

Fourth, recurrent whole‐genome duplication and subsequent diploidization and genomic downsizing across angiosperms may be a major force driving angiosperm species diversification. These processes provide the advantages of neofunctionalization and subfunctionalization (Leitch and Leitch, 2008; Jiao et al., 2011; Soltis and Soltis, 2012; Wendel, 2000, 2015; Dodsworth et al., 2016; Wong et al., 2020) while retaining both the traditionally envisioned benefits of genomic stability, sexual fertility, and balanced gene numbers (Stebbins, 1971; Soltis and Soltis, 1999; Conant et al., 2014; Wendel, 2015); and the photosynthetic advantages of a small cell size (Brodribb et al., 2013; Simonin and Roddy, 2018; Roddy, 2020; Théroux‐Rancourt et al., 2021) and—as argued here—help accelerate the evolution of plant anti‐herbivore defenses and thus speciation in angiosperms and specialized insect herbivores. Bursts of species diversification tend to follow, often with a substantial lag, whole‐genome duplications (Tank et al., 2015; Landis et al., 2018). The time required for subsequent diploidization, recovery of traditional and photosynthetic advantages, and plant–herbivore coevolution might account for much of that lag in diversification.

Finally, my model does not take account for the evolution of complex mixtures of defensive compounds. For example, Forrister et al. (2023) found an average of 194 ± 103 unique soluble chemical defensive compounds per species in *Inga*. Given the relatively low molecular weight of most of these (flavonoids and saponins), and the high rate of functional divergence expected, presumably species can diverge even more rapidly in multichemical space than they can in the defensive functions driven by any single compound. Unexpectedly, however, investment in the collection of these compounds was quite high (46% in young leaves, 24% in mature leaves), making them function as quantitative defenses despite the relatively small molecules involved in each compound (Wiggins et al., 2016). The defensive compounds in *Inga* depart from the expected qualitative defense‐low molecular weight‐low allocation–quantitative defense‐high molecular weight‐high allocation assumed in the model; this is likely to be true for many terpene‐based defenses, based on what is known about such defenses.

I believe that the most powerful tests of the hypotheses advanced here would include comparisons of genome size, vein density, photosynthetic rate, and nature of and allocation anti‐herbivore defense among many of the dozens of pairs of sister clades of angiosperms with high vs. low rates of net species diversification (see Magallón et al., 2019), as well as between these lineages and lineages within the ANA grade, Chloranthales, and magnoliids.

## AUTHOR CONTRIBUTIONS

**Thomas J. Givnish**: Conceptualization; Funding acquisition; Investigation; Writing—original draft.
